# The epidemiology and clinical spectrum of melioidosis in a teaching hospital in a North-Eastern state of Malaysia: a fifteen-year review

**DOI:** 10.1186/s12879-016-1583-2

**Published:** 2016-07-16

**Authors:** AbdelRahman Zueter, Chan Yean Yean, Mahmoud Abumarzouq, Zaidah Abdul Rahman, Zakuan Z. Deris, Azian Harun

**Affiliations:** Department of Medical Microbiology and Parasitology, School of Medical Sciences, Universiti Sains Malaysia, 16150 Kubang Kerian, Kelantan, Malaysia; Department of Orthopedic, School of Medical Sciences, Universiti Sains Malaysia, 16150 Kubang Kerian, Kelantan, Malaysia

**Keywords:** Melioidosis, B. pseudomallei, Pneumonia, Bacteremia, Malaysia, Dissemination

## Abstract

**Background:**

Over the last two decades, many epidemiological studies were performed to describe risks and clinical presentations of melioidosis in endemic countries.

**Methods:**

We performed a retrospective analysis of 158 confirmed cases of melioidosis collected from medical records from 2001 to 2015 in Hospital Universiti Sains Malaysia, Kubang Kerian, Kelantan, Malaysia, in order to update the current status of melioidosis clinical epidemiology in this putatively high risk region of the country.

**Results:**

Principal presentations in patients were lung infection in 65 (41.1 %), skin infection in 44 (27.8 %), septic arthritis/osteomyelitis in 20 (12.7 %) and liver infection in 19 (12.0 %). Bacteremic melioidosis was seen in most of patients (*n* = 121, 76.6 %). Focal melioidosis was seen in 124 (78.5 %) of patients and multi-focal melioidosis was reported in 45 (28.5 %) cases. Melioidosis with no evident focus was in 34 (21.5 %) patients. Fifty-four (34.2 %) patients developed septic shock. Internal organ abscesses and secondary foci in lungs and/or soft tissue were common. A total of 67 (41 %) cases presented during the monsoonal wet season. Death due to melioidosis was reported in 52 (32.9 %) patients, while relapses were occurred in 11 (7.0 %). Twelve fatal melioidosis cases seen in this study were directly attributed to the absence of prompt acute-phase treatment. Predisposing risk factors were reported in most of patients (*n* = 133, 84.2 %) and included diabetes (74.7 %), immune disturbances (9.5 %), cancer (4.4 %) and chronic kidney disease (11.4 %). On multivariate analysis, the only independent predictors of mortality were the presence of at least one co-morbid factor (OR 3.0; 95 % CI 1.1–8.4), the happening of septic shock (OR 16.5; 95 % CI 6.1–44.9) and age > 40 years (OR 6.47; 95 % CI 1.7–23.8).

**Conclusions:**

Melioidosis should be recognized as an opportunistic nonfatal infection for healthy person. Prompt early diagnosis and appropriate antibiotics administration and critical care help in improved management and minimizing risks for death.

## Background

Melioidosis occurs predominantly in Southeast Asia, northern Australia, the Indian subcontinent and China. Cases were first described in Burma 1912, Malaysia and Singapore 1913, Vietnam 1925 and Indonesia 1929. However, although Thailand and Australia have the highest endemicity of melioidosis, it was not documented in both countries before 1949 [1].

Melioidosis has emerged over the past 20 years as an important cause of morbidity, mortality, and fatal community-acquired bacteremic pneumonia. The clinical spectrum of melioidosis is broad; therefore, several clinical classifications were proposed [2]. *Burkholderia pseudomallei* strains differ in their ability to cause disease and the outcome depends on the immune status and response of the infected host. The incubation period is approximately 21 days which starts from the event of infection and extends until the onset of symptoms appearance. Nevertheless, the period has not been accurately estimated [3, 4].

Many of the cases of melioidosis occur in individuals with pre-existing illnesses; up to 80 % of melioidosis patients have one or more risk factor, whereas risk factors are less common in children [4]. The majority of recognised underlying risk factors in melioidosis patients are type II diabetes mellitus, chronic renal failure, and immunosuppressive treatments, in particular steroids [5]. The highly variable presentations of melioidosis make the clinical diagnosis impossible without the aid of confirmatory laboratory testing. Melioidosis should be suspected in cases of febrile illnesses presenting with respiratory failure, multiple subcutaneous lesions, or radiological pattern similar to that of tuberculosis, especially in diabetic patients, or those who resided or travelled to endemic areas [3]. As many saprophytes, *B. pseudomallei* is intrinsically resistant to many antibiotics including aminoglycosides and earlier generation cephalosporins [4].

In Malaysia, Deris et al. [6] had retrospectively studied bacteremic melioidosis in a tertiary hospital in Kelantan state from 2001 till 2005 and brought data on human risk factors and clinical manifestations. The present study was performed in the same hospital and updated the current status of melioidosis clinical epidemiology in that state by expanding the survey from 2001 to 2015 that covered all types of melioidosis.

## Methods

### Data collection & study population

The study was performed in Hospital Universiti Sains Malaysia by reviewing the clinical history of melioidosis patients over 15 years. This is an 800-bedded tertiary teaching hospital that is located in Kelantan, a northeastern state of Malaysia. Patients’ records were reviewed and analyzed for demographic data, clinical characteristics, management, complications and clinical outcome using a standardized checklist. The community population location for every patient was obtained from the address (the city and the state) at which the patient resided. Patient’s gender and mean age were reported and the age group was determined as (Infant ≤ 2 years); (>2 child ≤ 13 years); (>13 young adult ≤ 18 years); (>18 mature adult ≤ 39 years); (>39 middle-aged adult ≤ 59 years); and (old adult > 59 years) [7]. In addition, patients were clustered into two groups: less than 40 years and more than 40 years for association studies. The age grouping was done based on previous studies which noted that usually an individual started to acquire risk factors for melioidosis (particularly diabetes mellitus type 2) after the age of 40 [8, 9]. The race of patients was reported as Malay, Chinese, Indian or others.

### Environmental risks

As a part of environmental factors, the month of admission was recorded and assigned into two seasons: dry season (from the beginning of March until the end of October) and rainy season (from the beginning of November until the end of February). The exposure to environmental elements was also recorded during patients’ activity prior to admission, including farming, military work or recent travel to jungle or swimming in natural ponds and any other work.

### Clinical definitions

Clinical data were obtained from clinician notes as well as from laboratory records. All cases were previously confirmed by culture of *B. pseudomallei* from infected tissue, abscess aspirate, sputum, skin swab, blood, various kinds of body fluids or positive radiology in case of disseminated melioidosis with positive blood cultures. Melioidosis cases were specifically categorized into several grouping systems: acute versus chronic; bacteremic versus non-bacteremic or localized versus disseminated melioidosis. Bacteremic melioidosis was considered when the organism was isolated from blood culture. Recently, the infectious disease association of Thailand (IDAT) has classified melioidosis into four types: 1. bacteremic multifocal infection with septicemia 2. bacteremic localized infection with septicemia 3. non-bacteremic localized infection 4. and non-focal septicemia [2] based on clinical scenario of the disease and type of specimen that gave positive culture for *B. pseudomallei*.

In the present study, acute melioidosis was considered when the symptoms of confirmed melioidosis presented aggressively in the patient. If the duration of symptoms remained and did not resolve for more than two months, and then the infection was reported as chronic melioidosis. Recurrent infection was considered in case of the reappearance of melioidosis symptoms after the first episode treatment.

Clinical presentations included melioidosis pneumonia (or any lung infection such as empyema and bronchitis); infections of genitourinary (mainly prostate, urinary tract infection and kidneys); skin infection (cellulitis and intradermal abscess) and soft tissue infection (mainly tendons, ligaments, fascia, fibrous tissue and intramuscular deep seated abscesses), bone infection (includes osteomyelitis and septic arthritis), brain infection (cerebral abscesses, meningitis and encephalopathy), liver (abscesses and enlarged liver) and spleen (abscesses and enlarged spleen). Focal melioidosis was defined as clinical presentation of organ malfunction that was confirmed by laboratory tests and radiological investigation such as computed tomography (CT) scan. If the infection spread to other organs, thus it’s known as disseminated (or multi-focal) melioidosis. Estimated primary and secondary organ of involvement were determined according to the clinical event and progress of the disease. However, melioidosis with no evidence of organ involvement (non-focal) was reported as case of bacteremia with negative signs of organ burden. Culture-negative septic arthritis was reported as case of bacteremic melioidosis with clinical signs and symptoms of active arthritis presented with joint swelling, tenderness and inability to move. The anatomical location for all infections of skin, muscle, bone and joint were determined and marked. Melioidosis cellulitis of the foot was confirmed by swab and blood culture and was discriminated from diabetic foot cellulitis that was characterized by mixed poly-microbial infection.

Abscess, sepsis, severe sepsis and septic shock were also investigated as infection parameters. Sepsis was considered in case with temperature > 38 °C, pulse rate > 90 beats per minute, respiratory rate > 20 breaths per minute, pCO_2_ < 32 mm Hg, increased white blood cell count (>11 X 10^3^ cell/μl) and low blood pressure. Septic shock was reported in patients with sepsis and persistent unresponsive hypotension. Severe sepsis was identified as the presence of sepsis with markers of sepsis-related organ dysfunction.

A given case was considered as fatal melioidosis only when the death was reported as death due to melioidosis. In this case, death occurred within 72 h of blood culture positive for *B. pseudomallei* or clinical sepsis that did not improve after last positive blood culture. The effective fatal event of melioidosis was characterized as septic shock, severe sepsis, respiratory distress syndrome, cardiogenic shock or multi-organ failure.

Human risk factors were investigated individually for every patient including diabetes mellitus, chronic disease of the lungs or kidneys, immune suppression, that was either resulted from primary immune deficiency syndrome, viral infection or immune suppressants. Diabetes mellitus was considered according on clinician notes on patient’s history, serum glucose and glycosylated haemoglobin (HbA_1_C) results.

For statistical association study to examine the prediction for melioidosis outcomes, human risk factors were further grouped into a single statistical variable as the patient had any one of the mentioned biological risk factors.

Suspected co-morbid illnesses were reported and were associated with mortality outcome of melioidosis: they included co-infections with other pathogens like Gram negative bacteria, leptospirosis, hepatitis and human immunodeficiency virus, heart diseases, malignancies, haematological disorders and immune disturbances. Adequate treatment was reported when proper antibiotics were administered in accordance to the standard guideline.

### Statistical analysis

Statistical analysis was performed to investigate for frequencies among demographic data, clinical presentations and mortality. The descriptive analysis was presented as mean and standard deviations (SD) for normally distributed numerical variables. For categorical variables; all data were presented as frequency (n) and percentage (%).

For association statistical analysis, single and multiple logistic regression estimations were used between the dependent (outcomes of melioidosis) and independent (risk factors and co-morbids) variables. Moreover, simple logistic regression was used to identify the potentially risk independent variables associated with disseminated bacteremic melioidosis; melioidosis pneumonia and fatal melioidosis. Univariable analyses have examined: age ≥ 40 years old; age groups (pediatric, children, young adult, mature adult, middle-aged adult and old adult); diabetes mellitus; chronic kidney disease; immune suppression; cancers, general human risk; season on admission and exposure to environmental elements. Certain clinical illnesses were also included in some associations such as bacteremia, abscesses, acute infection, infections of the lungs, soft tissues, liver, spleen, osteoarticular tissue and brain. Co-morbid factors included microbial co-infections and chronic heart disease. Only independent variables with *p* value of less than 0.25 were selected and were further examined by multiple logistic regression. Results of regression were interpreted and presented as crude odds ratio, adjusted odds ratio, 95 % CI, and *p* value. Finally, conclusions were drawn based on the statistical results. For data entry and analysis, the Statistical Package for Social Sciences (SPSS, IBM, and Chicago, USA version 22.0) and STATA version 8.0 were used.

## Results

### Demography

The mean age of melioidosis patients was 45 (±19) years, ranging from 6 days to 83 years, with the majority of them were between 40 and 59 years of age. Forty two (26.6 %) patients were females and 116 (73.4 %) were males. Almost all subjects were Malay. Majority of patients 118 (74.7 %) came from the state of Kelantan, mainly from Kota Bharu, Bachok, and Pasir Mas; the rest were from Terengganu 37 (23.4 %), Kedah 2 (1.3 %) and one patient from Perak (0.6 %). The patients were exposed to various occupations that determined their contact with environmental elements. Although less than half of the patients were admitted during the rainy season throughout the study period, the highest individual frequency of monthly admissions was observed during November, December, January and February compared to admissions during other month over the year.

### Clinical data

Fever, infections of the respiratory system and soft tissues were commonly reported as the primary cause for emergency and hospital admissions. Bacteremic melioidosis was seen in 121 (76.6 %) patients, whereas focal melioidosis and disseminated infection were seen in 124 (78.5 %) and 45 (28.5 %) cases, respectively. Localized melioidosis predominated in both groups of melioidosis, bacteremic and non-bacteremic, while only five cases of disseminated melioidosis were non-bacteremic and restricted on skin and soft tissue infection. The vast majority of melioidosis cases (*n* = 150, 94.9 %) were considered acute infections, most of them (*n* = 116, 77.3 %) were with bacteremia. Similarly, out of eight cases of chronic melioidosis, the organism was isolated from the blood of five patients. All types of melioidosis were seen in all age groups, with gradual increased frequency with increased age and peaked at middle-aged group before declining at older age.

Among overall clinical presentations (initial and secondary), the lungs were the most frequently infected organs (*n* = 65, 41.1 %), followed by skin and soft tissues (*n* = 44, 27.8 %), bone and joints (*n* = 20, 12.7 %), liver (*n* = 19, 12.0 %), spleen (*n* = 15, 9.5 %) and brain (*n* = 9, 5.7 %). Genitourinary involvement was the least seen among the organs (*n* = 5, 3.2 %). Other unusual sites of infections were reported including parotid gland and lacrimal sacs (*n* = 4, 2.5 %). For all organs, the frequency of involvement became increased with the age; it means, no specific organ involvement was restricted to infancy, childhood and adolescent age groups.

Skin cellulitis, intradermal abscesses and intramuscular deep seated abscesses were reported in various anatomic locations (Table 1). In addition, tibial osteomyelitis was seen in two cases and septic arthritis of different joints was reported in 24 cases that were diagnosed either clinically or by culture (Table 2).

**Table 1:** Distribution of soft tissue abscesses and cellulitis throughout the body

Anatomic location	Skin and soft tissues^a^		
	Intradermal abscess *N* = 29	Cellulitis *N* = 23	Intramuscular abscess *N* = 17
Hand	1	2	
Foot	7	3	
Neck	5	1	
Leg		10	
Head	2	4	
Knee	1	1	
Shoulder	1		
Armpit	2		
Scrotum	2		
Chest wall	1		
Back	1		
Abdominal wall	2		
Face		1	
Forearm	4	1	2
Thigh			6
Calf			8
Gluteal			1

^a^A single patient may had several abscesses and/or cellulitis

**Table 2:** Bone and joint melioidosis in this study

Joint & bone location	Osteoarticular melioidosis^a^		
	Culture negative septic arthritis *N* = 8	Culture positive septic arthritis *N* = 16	Osteomyelitis *N* = 2
Elbow	1	4	
Hip		1	
Shoulder	3	1	
Knee	2	8	
Ankle	2	2	
Tibia			2

^a^A single patient may had several joints involvement

Sepsis, severe sepsis and septic shock were reported in 109 (69 %), 26 (16.5 %) and 54 (34.2 %) cases respectively. In addition to the expected primary organ involvement, subsequent clinically-evident secondary organ involvements were reported and examples are shown in Table 3.

**Table 3:** Secondary clinical foci for major primary diagnostic groups

Primary diagnosis	Total number	Number with secondary foci (% of total)	Most common secondary foci^a^
Pneumonia	59	30(50.8 %)	8 skin and soft tissues, 8 liver, 5 spleen, 5 brain and 4 osteoarticular
Skin & soft tissue infection	34	19(55.8 %)	5 lungs, 8 skin and soft tissue, 1 liver, 1 spleen and 4 osteoarticular
Liver infection	7	4(57.1 %)	3 spleen and 1 osteoarticular
Spleen infection	2	1(50.0 %)	1 liver
Osteoarticular infection	18	12(66.6 %)	2 lungs, 1 skin and soft tissue, 3 liver and 6 osteoarticular
Brain infection	2	1(50.0 %)	1 liver
Prostate infection	3	2(66.6 %)	1 skin and soft tissue and 1 liver

^a^More than single secondary focus found in some cases

Predisposing risk factors were reported in most of patients (*n* = 133, 84.2 %). Diabetes mellitus was the major predisposing factor in all but not children and young adults’ patients. Diabetes mellitus was present in 118 (74.7 %) patients. Other risk factors were immune disturbances (*n* = 15, 9.5 %), cancer (*n* = 7(4.4 %) and chronic kidney disease (*n* = 18, 11.4 %).

### Diagnosis and treatment

All cases were confirmed by cultivation of the organism from various clinical specimens. Only eight cases of septic arthritis were diagnosed clinically, serologically and by isolation of the organism from blood but not the synovial fluid. On admission, serology (IgM-indirect fluorescent assay and/or IgM/IgG-enzyme linked immunosorbent assay ELISA) was done for 50 patients and was positive for all, whereas the total white blood cell count ranged from 1.0 X 10^3^ cell/μl to 39.90 X 10^3^ cell/μl with the mean (± SD) of 13.85 X 10^3^ cells/μl (±6.67 X 10^3^ cells/μl)

Patients received empirical antimicrobial treatment prior to the availability of culture results, which was given based on the severity of the infection and suspected source of sepsis. In cases of high-grade fever, the empirical treatment comprised imipenem, ceftazidime, meropenem or piperacillin-tazobactam, while in less severe cases, in particular with suspected skin abscess origin, cloxacillin, cefotaxime, cefuroxime, metronidazole, azithromycin, fluconazole or ceftriaxone were administered. Standard melioidosis treatment was initiated upon culture and identification of *B. pseudomallei* results which were routinely available after 2 to 5 days. Intravenous ceftazidime 50 mg/kg (up to 2 g every 8 h) was given for infection with mild symptoms with no complications. In cases of severe acute infections and suspected dissemination, intravenous meropenem 25 mg/kg (up to 1 g every 8 h) or intravenous imipenem 20 mg/kg every 8 h were given. The duration of the first-line therapy was 10–14 days, which was extended to more than 4 weeks in cases of more severe disease. For eradication phase of treatment, oral trimethoprim/sulfamethoxazole (160 mg/800 mg 2 tablets every 12 h for 12–20 weeks) or amoxicillin/clavulanate (500 mg/125 mg; three tablets every 8 h for 12–20 weeks) were administered.

### Disease outcomes and co-morbidity

Death due to melioidosis was reported in 52 (32.9 %) of patients and recurrent infection occurred in four (2.6 %) patients. Twelve cases of fatal melioidosis cases seen in this study were directly attributed to the absence of prompt acute-phase treatment. Delayed admission, incomplete treatment course and missed and delayed diagnosis were frequently reported among patients with recurrent melioidosis. All patients with recurrent melioidosis succumbed to death in the last infection episode. Fifty one (32.3 %) patients suffered from other illnesses including co-infections (*n* = 17, 10.8 %), chronic heart diseases (*n* = 4, 2.5 %), immune disturbances (*n* = 15, 9.5 %), cancer (*n* = 7, 4.4 %) and chronic kidney disease (*n* = 18, 11.4 %). Among fatal cases, the disease ended with severe respiratory disease (*n* = 17), septic shock (*n* = 16), severe sepsis (*n* = 9), cardiogenic shock (*n* = 6), multi-organ failure (*n* = 3) and severe meningitis (*n* = 1).

### Association of risk factors with melioidosis

On simple logistic regression, neither human nor environmental risk factors were individually associated with disseminated septicemic melioidosis. However, when all human risks factors were grouped into a single independent variable, *p* value turned into statistically significant and the corresponding crude odd ratio and confidence interval became significant as well. On the contrary, the same analysis showed statistical and clinical significant associations of several individual clinical illnesses with disease dissemination. Multiple logistic regression analysis supported by model-fitness tests has defined the most probable risk factors that predicted the disease dissemination throughout body organs and tissues. Melioidosis patients had 4.36 times the odd to develop dissemination of the infection away from the site of origin (95 % CI 1.75, 10.84, *p* = 0.002) when adjusted for predisposing human risk factor. In addition, melioidosis patients had 13.83 times the odd to develop dissemination (95 % CI 2.26, 84.51, *p* = 0.004) when adjusted for bacteremia. However, other clinical conditions had increased the odd times for dissemination including lung melioidosis, skin and soft tissue melioidosis, osteoarticular infections and melioidotic abscesses (Table 4). In addition, patients with age ≥ 40 years had estimated risk for developing melioidosis pneumonia and fatal outcomes, whereas treatment has improved the mortality prediction of melioidosis (95 % CI 6.94, 12.63, *p* = 0.001) when adjusted for treatment (Tables 5 and 6).

**Table 4:** Factors associated with septicemic melioidosis with dissemination among 158 patients from multiple logistic regression analysis

Variable	Crude OR (95 % CI)^a^	Adjusted OR (95 % CI)^b^	Wald Statistics (df)	*p* value
*Human risk*				
Not presented	1.00			
Presented	4.37 (1.75, 10.81)	4.36 (1.75, 10.84)	10.05 (1)	0.002
*Bacteremia*				
Not presented	1.00			
Presented	3.16 (1.14, 8.73)	13.83 (2.26, 14.51)	8.01 (1)	0.004
*Lung melioidosis*				
Not presented	1.00			
Presented	3.38 (1.65, 6.95)	10.86 (4.14, 11.19)	13.54 (1)	0.001
*Skin and soft tissue melioidosis*				
Not presented	1.00			
Presented	3.42 (1.63, 7.20)	7.27 (1.39, 4.10)	5.52 (1)	0.019
*Osteoarticular melioidosis*				
Not presented	1.00			
Presented	8.05 (2.86, 22.71)	10.30 (8.62, 13.90)	17.71 (1)	0.001
*Melioidotic abscesses*				
Not presented	1.00			
Presented	4.59 (2.02, 10.40)	34.55 (7.64, 156.35)	21.15 (1)	0.001

- ^a^Simple logistic regression, ^b^Multiple logistic regression

**Table 5:** Factors associated with melioidosis pneumonia among 158 patients from multiple logistic regression analysis

Variable	Crude OR (95 % CI)^a^	Adjusted OR (95 % CI)^b^	Wald Statistics (df)	*p* value
*Age*				
Less than 40 years	1.00			
Equal to or more than 40 years	3.80 (1.72, 8.38)	3.44 (1.52, 7.82)	8.74 (1)	0.003
*Bacteremic melioidosis*				
Not presented	1.00			
Presented	6.30 (2.30, 17.24)	5.74 (2.06, 16.04)	11.12 (1)	0.001

- ^a^Simple logistic regression, ^b^Multiple logistic regression

**Table 6:** Factors associated with death due to melioidosis among 158 patients from multiple logistic regression analysis

Variable	Crude OR (95 % CI)^a^	Adjusted OR (95 % CI)^b^	Wald Statistics (df)	*p* value
*Age*				
Less than 40 years	1.00			
Equal to or more than 40 years	2.48 (1.09, 5.64)	6.47 (1.79, 23.38)	8.11 (1)	0.004
*Septic shock*				
Not presented	1.00			
Presented	14.00 (6.22, 31.50)	16.55 (6.10, 44.90)	30.34 (1)	0.001
*Co-morbidity*				
Not presented	1.00			
Presented	2.25 (1.11, 4.55)	3.06 (1.12, 8.41)	4.72 (1)	0.030
*Anti-melioidosis therapy*				
Not Given	1.00			
Given	35.03 (4.40, 27.66)	16 (6.94, 12.63)	11.64 (1)	0.001

^a^Simple logistic regression, ^b^Multiple logistic regression

## Discussion

We explored the clinical characteristics of melioidosis in the Northeastern part of Malaysia and have updated the literature databases with latest statistics related to the disease. In this study, melioidosis was frequently reported among middle-aged patients, as reported in other surveys [8–10] that linked the reason with the acquiring of risk illnesses with aging. For example, type two diabetes mellitus is the prominent risk factor for melioidosis and is usually newly diagnosed in mature adults, which in turn will increase the risk of the infection. On the contrary, children, adolescent and young adult age groups have minor infections probably due to relative lack of predisposition. However, a few epidemiological surveys and case reports of melioidosis in these age groups have been published in Malaysia and elsewhere [11–19].

In this study, males were more risky for acquiring the infection than females; probably because of the differences in exposure to bacterial reservoir such as soil during rice farming and inhalation of contaminated dust. The male-to-female ratio for melioidosis was higher in all studies performed in Malaysia, Australia, and Singapore [8, 9, 20, 21]. The vast majority of patients were Malay, since the patients came primarily from different locations in Kelantan state, as well as from Kuala Besut, Terengganu. Both states are predominated by Malay race population. Same findings were reported by Hassan et al. [9] who analysed data of 129 Malay patients in Kedah, a state that resembles Kelantan in ethnic demography.

Farming, military works and other occupational activities were reported. Although limited data were collected about the patients’ occupation due to missing of records, environmental exposure was expected since the majority of patients were originated from rural and agricultural regions in Kelantan. In Kedah state, farming, fishing and forestry were common occupations for admitted patients [9]. In Thailand 81 % of melioidosis cases were diagnosed in rural rice farmers and their children [22], whereas in Singapore, melioidosis has occurred in construction workers, gardeners and military personnel [20]. Similarly, 75 % of melioidosis cases in Northern Australia were associated with recreational activities resulting in exposure to environmental *B. pseudomallei*, whereas 18 % of the cases had clear occupational exposure [8].

Months from November till February represent the rainy season in the eastern coast of Peninsular Malaysia in which extreme weather changes happen including flooding. This may facilitate dynamic distribution of *B. pseudomallei* in the environment and thus increasing bacterial infective load in surface water and soil. Moreover, epidemiologic data suggested that massive volumes of rainfall result in raising the water level on land, which leads to the accumulation of *B. pseudomallei* on surface soil and becomes a reservoir for inhalation of aerosolized bacteria for human and animal. In addition, bacterial cells might be carried far away from their reservoirs to contaminate water sources and farms [23–25]. Recently, an outbreak of melioidosis was documented during the flood that hit Malaysia in December 2014 in which 20 cases were confirmed in the affected places. Another post-flood melioidosis report by Pahang state health officials documented 21 melioidosis cases detected during the first 2 months of 2015, in comparison to 16 cases throughout 2014 (http://www.worldwideoutbreak.com). In Kedah, melioidosis cases and deaths were highest in the wettest months in Kedah state [9]. In general, the correlation between melioidosis and rainfall is much stronger in northeast Thailand and northern Australia that experience, like Kelantan state, an intensely wet and windy season, and a dry season with very little rain [23, 26].

However, these findings disagreed with those obtained from Kuala Lumpur state and Singapore reports that suggested no association between rainfall intensity and melioidosis; but instead, it was attributed to decreased environmental and agricultural areas. In addition, the rainfall is relatively even along the year with no distinct wet or dry seasons [25, 27]. In contrast, a recent report from Singapore reviewed 550 melioidosis cases along a 10-year period in a highly urbanized city had found an association of rainfall and humidity levels with disease incidence. It was suggested that water, rather than soil, might be the central vehicle for transmission and acquisition of this disease, in particular, most patients in this study did not report occupational or recreational exposure to soil [28]. The assumption from that study might be supported by a recent Thai matched case–control study and other studies from Australia which suggested that acquisition of *B. pseudomallei* after ingestion of unchlorinated domestic water supplies might be more common than previously thought [29–31].

In this study, melioidosis pneumonia and soft tissue infections were repeatedly seen. In general, fever and respiratory symptoms are well characterized manifestations that require urgent admissions. In addition, abscess-induced swelling with tenderness was the most common primary presentation of soft tissue melioidosis that might be accompanied with fever in case of dissemination [8]. However, the infection acquisition route may determine the primary manifestations of disease. Inhalation of aerosolized dust and percutaneous bacterial inoculation are the most common infection route that resulted in higher frequencies of pneumonia and skin and soft tissue abscesses and cellulitis, respectively [2].

Acute bacteremic melioidosis with organ involvement was seen in most of patients. Majority of melioidosis surveys focused on bacteremic melioidosis due to its higher frequency [6, 8]. Although dissemination occurs after hematogenous spread of bacteria from one organ to another, five cases of disseminated soft tissue melioidosis were reported in this study without isolation of bacteria from blood samples probably because of the close distances between the infected foci.

Melioidosis was seen in all age group, particularly at the middle age. However, McLeod et al. [15] reported increased frequency of non-bacteremic localized melioidosis in age group less than 16 years and attributed that difference to lack of risk factors in this age group that induce bacteremia and dissemination.

On the other hand, septic arthritis, intra-organ abscesses of brain, spleen, liver, prostate and bone tissue were more attributed with secondary hematogenic spread of bacteria from the original infected foci. However, some cases of septic arthritis were secondary to adjacent intradermal abscesses [32]. Septic arthritis and osteomyelitis due to *B. pseudomallei* are rare but still considered well-recognized presentations of melioidosis [33]. In the present study, only 20 cases of osteoarticular involvement were reported over 15 years review, which concurs with other previous surveys [6, 8]. The reason behind the low-rate involvement of osteoarticular tissue in melioidosis might be attributed to the low blood supply to these locations comparing to other organs and, in parallel, the fact that the majority of melioidosis cases are bacteremic and affect mostly organs rich with blood supply such as liver, spleen, lungs and brain [34, 35]. In another study performed in tropical Australia, the percentages of primary and secondary melioidotic septic arthritis among 536 confirmed cases were 2.4 % and 2.6 %, respectively [36].

Cellulitis and abscesses were reported in various anatomic locations without predominance in one location over another and were either singly or secondary to each other or to different primary infected foci. This pattern of infection distribution reflected the unspecific behavior of the bacteria during its pathogenesis toward certain anatomic location. Same infection pattern was observed for septic arthritis but with predominance of infection of the knee. In a study done by Pui and Tan [37] the knee was the most common infected location followed by ankle, foot, shoulder and hip; results agree with our findings and other reports [32, 36].

In contrast to adult melioidosis, it was reported that non-bacteremic melioidosis associated with skin and soft tissue was more frequent in patients less than 16 years old rather than bacteremic pneumonic melioidosis [15]. In this study, melioidosis among this age group did not show disease manifestations that differ from those in adults, which might be due to low number of reviewed patients under this age category to enable adequate discrimination.

Unlike reports from northern Australia [38] and Thailand [22], genitourinary and parotid melioidosis were the least seen among the infected organs in this study. It was postulated that there are regional difference in melioidosis presentation that was either due to host, bacterial, or environmental factors [39]. However, in spite of intensive previous works to discover the fact behind regional difference in melioidosis clinical presentations, conclusions have been difficult to make from relatively small sample sizes and then reasons for this difference remain unclear [40, 41]. Most of the less common presentations of melioidosis seen in this study have also been described in other reports including parotid gland [42] and lacrimal sac [43].

Dissemination of the infection to other organs might lead to microbial colonization inside deep seated abscess that interfere with normal organs functions [44]. In the present study, approximately 60 % of primary lung, soft tissue and osteoarticular melioidosis cases exhibited dissemination to secondary organs. In general, whatever the primary clinical presentation of melioidosis was, secondary focal dissemination is common, presumably from bacteremic spread, which was reported in 76.6 % in our patients. Secondary pneumonia, soft tissue and septic arthritis were also common [8]. Musculoskeletal melioidosis is rare but not uncommon; septic arthritis and osteomyelitis are usually secondary infections commonly occur following bacterial dissemination from infection elsewhere in the body. To a lesser extent, septic arthritis or osteomyelitis can be the primary manifestation of melioidosis. The commonest musculoskeletal melioidosis presentations are soft tissue involvement, followed by osteomyelitis, septic arthritis and, less common, spine infection [36].

Melioidosis occurs commonly in individuals with pre-existing illnesses; up to 80 % of melioidosis patients have one or more risk factors, whereas risk factors are less common in children [11, 15]. In our study, predisposing risk factors were reported in most of patients. Type two diabetes mellitus was the major predisposing factor in all but not children and young adults’ with nearly all had chronic uncontrolled type 2 diabetes. In Malaysia, the rate of diabetes mellitus in patients with bacteremic melioidosis was 56 % [9]. Another reported risk factor was immune disturbance due to cancer or autoimmunity medications, primary immune deficiency condition or chronic kidney disease. However, 25 (15.8 %) of patients did not have any reported predisposing factor. In Australia, both hazardous alcoholism and type 2 diabetes mellitus were equally reported as frank risk factors for melioidosis in 39 % of patients and showed similar mortality rates in Australia [8], whereas only type 2 diabetes mellitus was the predominant predisposing factor in Malaysia [6, 9, 45].

In this study, mortality rate was 32.9 %. The mortality rate of acute melioidosis was stated as 19-40 % in endemic countries including Singapore, Northern Australia, northern Thailand and Malaysia [9, 20, 30]. Untreated bacteremic melioidosis increases the mortality rates upon hospital admission [46]. In the present study, the absence of prompt acute-phase treatment was common cause of death in some cases, which might be related to delayed admissions of severe cases that lead to fatal outcomes despite the administration of standard empirical treatment. In addition, some cases with fever and skin abscess were treated empirically for suspected Gram positive or fungal sepsis. This study reported 13 patients who received only empirical treatment that might have failed to save their lives. Deris et al. [6] and other literatures have recommended urgent laboratory diagnosis and increased level of melioidosis suspicion to improve the management and reduce mortality.

In the present study, recurrent infections were attributed to delayed admission, incomplete treatment course and missed or delayed diagnosis. In Thailand, the overall recurrent infections were up to 30 % per year of survivors who had severe melioidosis [47]. While in Malaysia, the rate of recurrence was approximately 13 % over a period of 5 years [3]. Early hospital admission, prompt diagnosis and treatment and completion of medication course will reduce recurrent infections [4]. Four patients had several recurrent infections episodes and all succumbed to the infections. For some patients, disease pattern and bacterial genotype were not similar during the different episodes of the infections which suggest diversity of bacterial genotypes in a given geographical area. It was reported that recurrent infection occurs in 6–13 % of melioidosis cases due to relapse of the infection by the same bacterial strain rather than reinfection with a new bacterial strain, especially when occurring within a year of primary infection [48]. In a study, 140 patients with recurrent melioidosis showed similar patterns of disease occurred during different disease episodes, regardless of whether this was caused by relapse or re-infection [49]. Chronic co-morbid illnesses are common in melioidosis patients and worsen the clinical presentation of the disease and might be influenced with mortality [2].

The present study has defined the probable risk factors associated with disseminated and lung melioidosis and fatal outcomes. Among the considered risk factors, diabetes mellitus is responsible for abnormal increased blood sugar, whereas chronic immune syndromes compromise the circulating immunity; together, both conditions facilitate bacteremia, which induce haematogenous spread of bacteria away from the origin of the infection [50]. The association of diabetes with bacteremic melioidosis has been noted in Thailand [24]. Lungs and soft tissues are common sites of infection and are well connected with capillary networks for blood supply due to their vital functional role for the body; this physiological role may expose these organs for the infection via circulating bacteria. In addition, *B. pseudomallei* experience chronic persistence inside osteoarticular tissues upon colonizing various organs and tissues and stay inside cysts and abscesses that represent bacterial source and reservoir from which the bacteria may spread and get access to other organs [4]. In Australia, age, diabetes, alcoholism and chronic illness were associated with bacteremic melioidosis [8], whereas diabetes mellitus was the predominant associated risk factor with bacteremic melioidosis in the same study area [6].

In this study, melioidotic bacteremia and age ≥ 40 years were each associated with a propensity for presentation with pneumonia in comparison to other presentations. Currie et al. [8] had stated risk factors associated with melioidosis pneumonia; on simple logistic regression age ≥ 50 years and diabetes mellitus showed significant increase in the odd for developing pneumonia. However, multiple logistic analyses reported the association of chronic illnesses, alcohol and Kava consumption with melioidosis pneumonia.

In this study, age ≥ 40 years, septic shock and co-morbid illnesses were independent predictors for mortality. In addition, anti-melioidosis treatment had improved protection against fatal disease outcomes. In Australia, only age ≥ 50 years and pre-disposing risk were associated with mortality [8], whereas in Malaysia, patients with diabetes suffered significantly more mortality from melioidosis, this relationship was more influenced by age [9]. Another report from Kuala Lumpur, Malaysia had studied the predictors of severe disease or death for 85 melioidosis patients and stated that male patients with age more than 40 years old and diabetes mellitus were the most significantly associated with bad prognosis and death; severe disease or death occurred in 28 (32.9 %) of cases [51]. Septic shock is the normal pathological consequence following bacterial colonization in blood vessels; in this study, sepsis was reported in 69 % of bacteremic melioidosis cases that in turn increase the chance for fatal septic shock. Again, the increase in the age has led to worsen disease events, prognosis and outcomes due to presence of predisposing factors that may become life-threatening co-morbid illnesses. Treatment outcomes are somewhat satisfactory which were attributed to prompt administration of the proper antibiotic in majority of cases as well as the absence of carbapenem-resistant strains of *B. pseudomallei* in the area of the study [45, 52–54].

## Conclusion

This study has provided a major review for melioidosis among selected population clusters residing the Northeastern part of Peninsular Malaysia. Diabetes mellitus is the major risk factor for bacteremic melioidosis, which is the most common type of the infection. Thus efforts toward disease prevention may include evading conditions that predispose melioidosis by controlling, for example, food habits and life style associated with diabetes and provide complete management for diabetic patients to reduce its incidences. These precautions must target age group more than 40 years old due to increased disease risk in this age stage. In addition, advices must be provided to people in both rural and urban areas regarding water hygiene and precautions must be made during rainy seasons by increasing the level of suspicion of melioidosis in the medical wards and to minimize personal contact with environmental elements to reduce their exposure to contaminated particles.

Disease management in this study has successfully reduced the incidence of undiagnosed fatal melioidosis to minimum, however, still the ambition for further reducing the incidence by the addition of rapid point-of-care testing for melioidosis to provide prompt diagnosis and selective treatment. For clinical epidemiology study, more cases could be further reviewed from other hospitals and medical institutes throughout Malaysia to provide complete picture for melioidosis status that may uncover new correlations and insights for further understanding of the disease and improve management.

## Abbreviations

ELISA, enzyme linked immunosorbent assay; IDAT, infectious disease association of Thailand
